# AAV production in stable packaging cells requires expression of adenovirus 22/33K protein to allow episomal amplification of integrated rep/cap genes

**DOI:** 10.1038/s41598-023-48901-z

**Published:** 2023-12-07

**Authors:** Weiheng Su, Leonard W. Seymour, Ryan Cawood

**Affiliations:** 1 Department of Oncology, University of Oxford, Old Road Campus, Oxford, OX3 7DQ UK; 2 OXGENE Ltd, Oxford Science Park, Oxford, OX4 4GA UK

**Keywords:** Biotechnology, Genetics, Molecular biology

## Abstract

Efficient manufacture of recombinant adeno-associated virus (rAAV) vectors for gene therapy remains challenging. Packaging cell lines containing stable integration of the AAV rep/cap genes have been explored, however rAAV production needs to be induced using wild-type adenoviruses to promote episomal amplification of the integrated rep/cap genes by mobilizing a *cis*-acting replication element (CARE). The adenovirus proteins responsible are not fully defined, and using adenovirus during rAAV manufacture leads to contamination of the rAAV preparation. ‘TESSA’ is a helper adenovirus with a self-repressing Major Late Promoter (MLP). Its helper functions enable efficient rAAV manufacture when the rep and cap genes are provided *in trans* but is unable to support rAAV production from stable packaging cells. Using rAAV-packaging cell line HeLaRC32, we show that expression of the adenovirus L4 22/33K unit is essential for rep/cap amplification but the proteins are titrated away by binding to replicating adenovirus genomes. siRNA-knockdown of the adenovirus DNA polymerase or the use of a thermosensitive TESSA mutant decreased adenovirus genome replication whilst maintaining MLP repression, thereby recovering rep/cap amplification and efficient rAAV manufacture. Our findings have direct implications for engineering more efficient adenovirus helpers and superior rAAV packaging/producer cells.

## Introduction

Adeno-associated virus (AAV) based vectors are attracting substantial interest for gene therapy to correct or supplement defective genes and to deliver therapeutics, such as antibodies, that improve disease phenotypes^1–3^. Recombinant AAV (rAAV) vectors have been administered in over 300 clinical trials with three gene therapy drugs approved by the US Food and Drug Administration (FDA) in clinical use (clinicaltrials.gov, 16.02.2023)^4^. Efficient and scalable manufacture of high-quality rAAV to support preclinical, and clinical trials, remains a significant challenge. The AAV replicative life cycle requires the presence of a helper virus, such as adenovirus, to provide essential factors for regulating AAV gene expression and DNA replication^5^. rAAV vectors are missing the rep and cap genes from the AAV genome, essential for viral DNA replication and capsid formation, to make space for a transgene expression cassette. Only the viral inverted terminal repeats (ITRs) are retained in the transfer vector, palindromic sequences that confer a DNA hairpin structure required *in cis* for replication and encapsidation of the single-stranded viral genome^6,7^. The use of stable rep/cap cell lines, generally based on HeLa cells, has been explored extensively as a scalable packaging system for rAAV manufacture. In this approach, the rep and cap genes are integrated into the cell’s chromosome, and the rAAV transfer genome may be similarly integrated or can be delivered by plasmid transfection or infection with a hybrid Adenovirus-AAV vector. In each case, the production of rAAV is induced by infection with wildtype adenovirus to provide specific ‘helper’ functions^8–10^.

An important and unique characteristic of stable rep/cap packaging cell lines is the episomal amplification of the integrated rep and cap DNA, up to 1000-fold, after infection with wildtype adenovirus. This allows the production of sufficient Rep and Cap proteins for efficient replication of the rAAV genome^11^. Amplification of rep/cap DNA is crucially dependent on a *cis*-acting replication element (CARE), mapped to the p5 promoter of the rep gene, and is known to require DNA binding protein (DBP) from adenovirus alongside the AAV Rep proteins^12^. However, transfection of the adenovirus genome DNA into these stable packaging cells is unable to induce CARE-dependent amplification, indicating that currently-undefined factor(s) present during adenovirus infection but not during adenovirus genome transfection play a role in mobilizing the CARE element^8,13,14^.

We recently described a new helper adenovirus system entitled ‘Tetracycline-Enabled Self-Silencing Adenovirus’ (TESSA) wherein the adenovirus Major Late Promoter (MLP) was modified in situ to enable self-repression of promoter activity and truncate the adenovirus replication cycle for contaminant-free manufacture of rAAV^15^. While TESSA was fully capable of providing helper functions to enable rAAV replication when the rep/cap genes were provided *in trans* via plasmid transfection or delivered using the adenoviral vector, here we observed that repression of the adenovirus MLP from TESSA inhibited rep/cap amplification and rAAV production from stable rep/cap model cell line, HeLaRC32. Using a series of siRNA and complementation assays, we present evidence that the adenovirus late proteins, 22K/33K, which share a common N terminal 105 amino acid sequence, are necessary for efficient replication of the rep/cap genes integrated within the packaging cells but can be titrated away by DNA replication of adenoviral genomes. 22K/33K proteins are multifunctional and involved in regulating the expression of early and late adenoviral genes, RNA splicing, and the genome encapsidation process^16,17^. During adenoviral infection, 22K/33K proteins are initially expressed at low levels under the control of the L4 internal promoter (L4P), but are expressed at much higher levels from the MLP later in infection^18,19^. Taken together, our results suggest a model wherein, normally, high levels of 22K/33K are expressed from the activated MLP to support both adenovirus and rAAV replication in stable rep/cap packaging cell lines, but repression of the MLP from TESSA restricted the amount of 22K/33K proteins available to support rep/cap amplification. Interestingly, low levels of 22K/33K produced from the L4P appear sufficient to allow some rep/cap amplification and rAAV replication, but only if adenovirus genome replication is inhibited to prevent sequestration of 22K/33K by the newly produced genomes. We demonstrate this effect by inhibiting adenovirus polymerase activity. Both siRNA knockdown of the adenovirus DNA polymerase or use of a temperature-sensitive DNA polymerase mutant in TESSA (TESSA-tsDNA) significantly decreased adenovirus genome replication and recovered rep/cap gene amplification for efficient rAAV production.

## Results

### CARE-based amplification and AAV production from Rep/Cap packaging cell line (HelaRC32) depends on MLP activity

**Figure 1:**
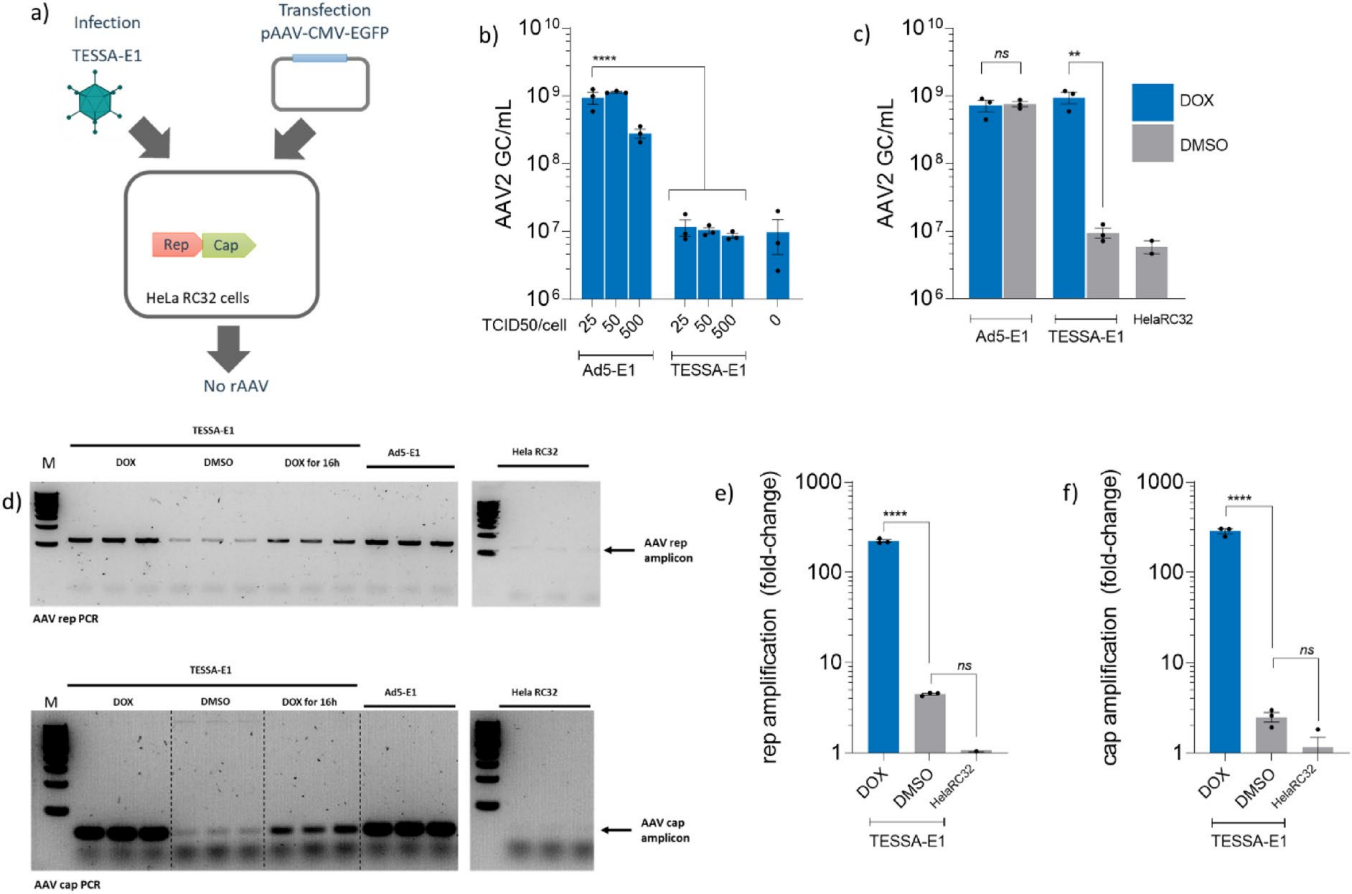
Repression of the adenovirus MLP inhibited rAAV2 production from HelaRC32 cells. (**a**) Schematic diagram for production of rAAV2 from HelaRC32 cells. Cells were transfected with the plasmid pAAV-EGFP and infected with Ad5-E1 or TESSA-E1 at the indicated multiplicity of infection (MOI). (**b**) Production of rAAV2 from HelaRC32 cells. rAAV2-EGFP were harvested at 96 h post-infection (hpi) and quantified by EGFP-specific qPCR. Analysed by one-way ANOVA followed by Tukey post hoc test comparing TESSA-E1 versus Ad5-E1. ****p ≤ 0.0001. (**c**) Production of rAAV2-EGFP using Ad5-E1 or TESSA-E1 (MOI of 50 TCID50/cell) from HeLaRC32 cells cultured with DMSO or doxycycline. Data presented as mean ± SD of triple biological replicates. Analysed by one-way ANOVA followed by Tukey post hoc test comparing doxycycline versus DMSO. **p = 0.0017. (**d**) Episomal DNA amplification in HeLaRC32 cells. Cells were infected with Ad5-E1 or TESSA-E1 and cultured with DMSO or doxycycline and used for PCR with AAV2 rep (top blot) and cap-specific (bottom blot) primers and resolved by gel electrophoresis. Cropped images show control amplicon detection by PCR using AAV2 rep and cap primers from HeLaRC32 cells, resolved in respective gel electrophoresis. The uncropped blots are presented in supplementary Fig. S1 [and supplementary information file S1]. Data presented as n = 3. DNA from HeLaRC32 cells infected (MOI of 50 TCID50/cell) with TESSA-E1 and cultured with DMSO or doxycycline was quantified by (**e**) rep or (**f**) cap-specific qPCR. Data presented as fold change above non-infected HeLaRC32 cells. Data presented as mean ± SD of triple biological replicates. Analysed by one-way ANOVA followed by Tukey post hoc test comparing doxycycline versus DMSO. ****p ≤ 0.0001.

TESSA is a new self-inhibiting adenovirus helper that produces large quantities of highly infectious rAAV vectors that can be used alongside transfection of the rep/cap plasmids and rAAV genome in HEK293 cells. Previous studies have shown significant increases in the yield of infectious AAV particles compared to helper-free systems, with negligible adenovirus contamination at pre-purification^15^. On this basis we anticipated TESSA would show good helper activity in AAV packaging cells, wherein rep/cap is integrated into the cell genome. Because HeLaRC32 cells do not provide the adenovirus E1 genes that are required for AAV replication, we engineered E1 back into TESSA (TESSA-E1) and also into an E1/E3-deleted Adenovirus type 5 vector (to yield Ad5-E1), and provided rAAV vector (pAAV-EGFP) by plasmid transfection (Fig. 1a). However, as shown in Fig. 1b, while the Ad5-E1 helper was able to support high-titre rAAV production, yielding ~ 1 × 10^9^ genome copies (GC) per mL of rAAV2-EGFP, TESSA-E1 was unable to produce rAAV with levels detected comparable to qPCR background control. TESSA-E1 is designed to have the MLP normally inactive, but inducible following addition of doxycycline to allow the adenovirus to complete its full life cycle. This is important to enable production of stocks of TESSA during adenovirus manufacture, but not necessary during rAAV production and allows TESSA to provide ‘helper’ functions without adenovirus contamination. Intriguingly, addition of doxycycline to TESSA-E1 during rAAV production in HeLaRC32 packaging cells completely restored the rAAV yield, achieving similar levels to those shown using the Ad5 helper virus (Fig. 1c). These data suggest that adenovirus factor(s) that is expressed under transcriptional control of the MLP is necessary for efficient rAAV production from the rAAV packaging cell line. We next explored whether the beneficial effect of MLP activation on rAAV production was associated with episomal amplification of the integrated rep/cap gene. As shown in Fig. 1d, in a production run using TESSA-E1 in the absence of doxycycline (where the MLP is turned off) there was only a very weak PCR signal detected for both AAV2 rep and cap genes, but when doxycycline was added (and the MLP activated) a strong PCR band was detectable for both genes. Interestingly, in TESSA-E1 infected cells and treated with doxycycline for 16 h, PCR of rep and cap bands of intermediate intensity can be observed suggesting sustained transcription from the MLP may be essential for maintaining rep and cap DNA amplification. These results were confirmed by AAV2 rep and cap specific qPCR, which showed over 200-fold amplification of integrated rep (Fig. 1e) and cap (Fig. 1f) DNA from HeLaRC32 cells following infection with TESSA-E1 in the presence of doxycycline. In the DMSO treated samples, a two- to fourfold increase in rep and cap DNA was observed above non-infected HeLaRC32 cells. Overall, these results suggests that episomal rep and cap amplification is dependent on expression of an adenovirus late protein(s) although precisely which protein(s) remained unknown.

### rAAV production in HeLaRC32 cells from TESSA-E1 can be restored using supplementary Ad5-E1

**Figure 2:**
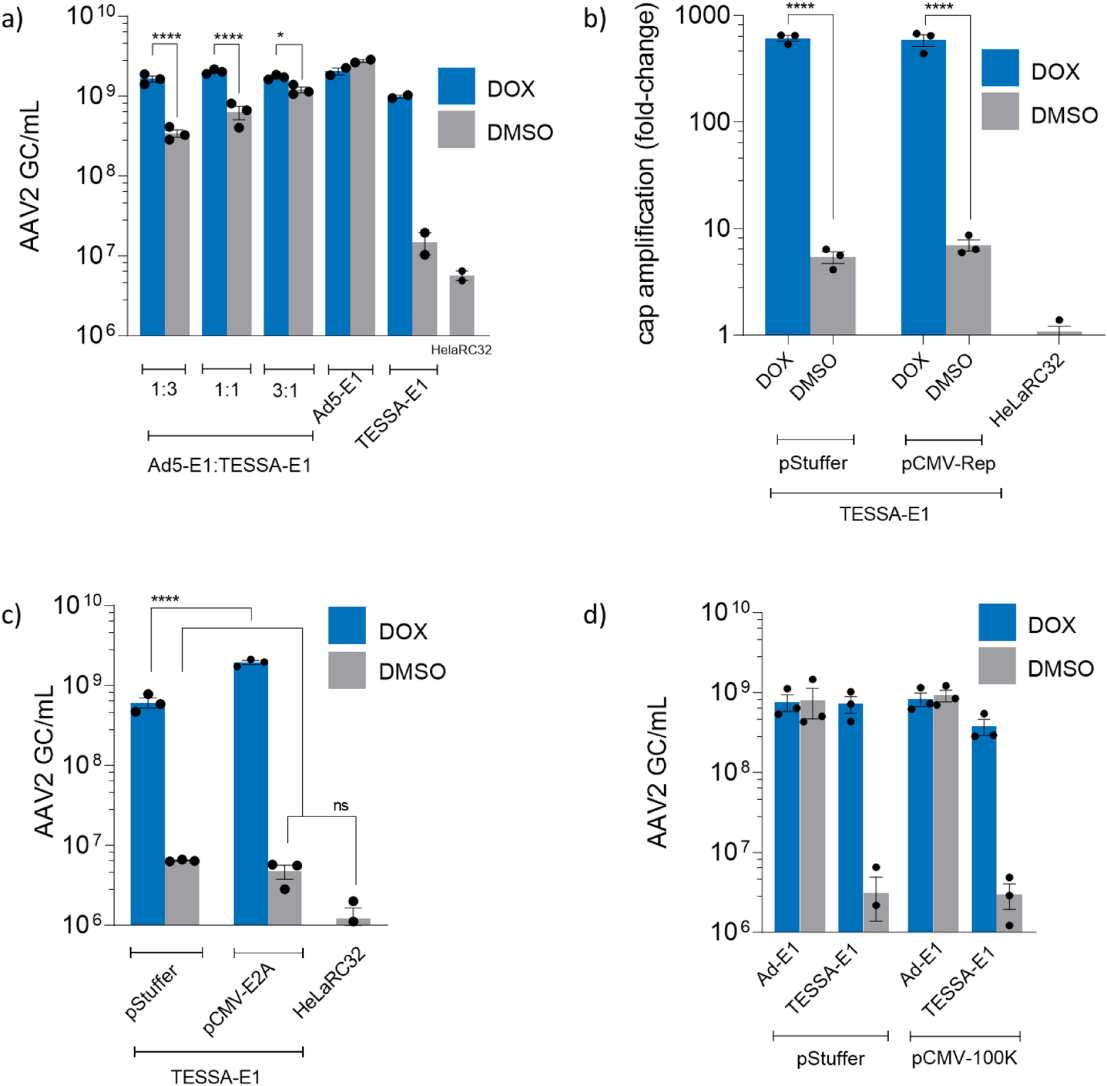
Expression from the adenovirus MLP is required to maintain rep and cap DNA amplification from HeLaRC32 cells. (**a**) Production of rAAV2-EGFP via co-infection of TESSA-E1 with Ad5-E1. HeLaRC32 cells were transfected with pAAV-EGFP and infected (total 50 TCID50/cell) with Ad5-E1, TESSA-E1, or co-infected at the indicated ratio. (**b**) HeLaRC32 cells transfected with pCMV-Rep or pStuffer and infected with TESSA-E1. Cells were supplemented with doxycycline or DMSO. Total DNA extracted at 72 hpi was quantified by AAV2 cap-specific qPCR. Data presented as fold change above non-infected HeLaRC32 cells. rAAV2-EGFP production from HelaRC32 cells were co-transfected using pAAV-EGFP with pCMV-E2A DBP (**c**) or pCMV-100K (**d**) and infected with TESSA-E1 or Ad5-E1, as indicated, and supplemented with DMSO or doxycycline. DNAse-resistant particles were quantified at 96 hpi by EGFP-specific qPCR. Data presented as n = 3 (mean ± SD). Analysed by one-way ANOVA followed by Tukey post hoc test. *p ≤ 0.05, ****p ≤ 0.0001.

To determine whether the factor(s) restricting the ability of TESSA-E1 to produce rAAV with its MLP repressed in HeLaRC32 cells were dominant or recessive, we added different ratios of Ad5-E1 and TESSA-E1 (total MOI of 50 TCID50/cell in each case) to HeLaRC32 cells. As shown in Fig. 2a, in each case the yields of rAAV in the absence of doxycycline were roughly proportionate to the amount of Ad5-E1, indicating that the E1-expressing Ad5 can work perfectly well in the presence of MLP-inactive TESSA-E1. The production yield of rAAV from infection with the Ad5-E1 vector was measured at ~ 2.5E+9 GC/mL, an approximate twofold vector titre increase compared to previous production runs (Fig. 1b,c), reflecting the variations between different batches of rAAV productions. We then assessed whether addition of exogenous AAV Rep protein would rescue the ability of TESSA-E1 (MLP-inactive) to enable CARE-amplification, supposing that the helper activity of TESSA-E1 may be limited by failure to induce sufficient expression of Rep from the HeLaRC32 cells. Transfection of plasmid expressing AAV2 Rep failed to recover CARE-amplification (determined by measuring replication of AAV cap DNA) by TESSA-E1 in the absence of doxycycline (Fig. 2b). Consequently, amplification of AAV rep DNA from the HeLaRC32 cells is also not expected as rep and cap amplified as a contiguous DNA sequence molecule^11,13^, this finding suggests that the missing late protein(s) play a role alongside Rep in mobilising the CARE element. Similarly, we supposed the available levels of E2A DBP might be limiting because as the TESSA-E1 genome replicates it may, potentially, sequester large amounts of DBP. However, transfection of an adenovirus DBP expression plasmid failed to recover rAAV production when the MLP is repressed (DMSO group) in TESSA-E1, although a 70% increased rAAV yield was observed in the presence of doxycycline, suggesting that an increase in DBP may enhance rAAV replication (Fig. 2c).

Recently, it was shown that an adenovirus deleted of 810 bp within the L4 100K gene failed to promote amplification of the stably integrated rep/cap genes, and high titre rAAV production from an A549-based stable AAV packaging cell line^20^. As the 100K protein is expressed from the MLP, we assessed whether it comprises the missing ‘late’ adenovirus protein from TESSA-E1, during MLP repression, that is required to facilitate rep/cap DNA amplification^21^. We therefore co-transfected HeLaRC32 cells with a plasmid expressing the Ad5 100K from the CMV promoter alongside pAAV-EGFP, and infected with TESSA-E1 or Ad5-E1. As shown in Fig. 2d, expression of the 100K was also unable to recover rAAV production or rep/cap amplification (supplementary Fig. S2) in the absence of doxycycline.

### Expression of the intermediate and late L4 22/33K unit is required for rAAV production from HeLaRC32 cells

**Figure 3:**
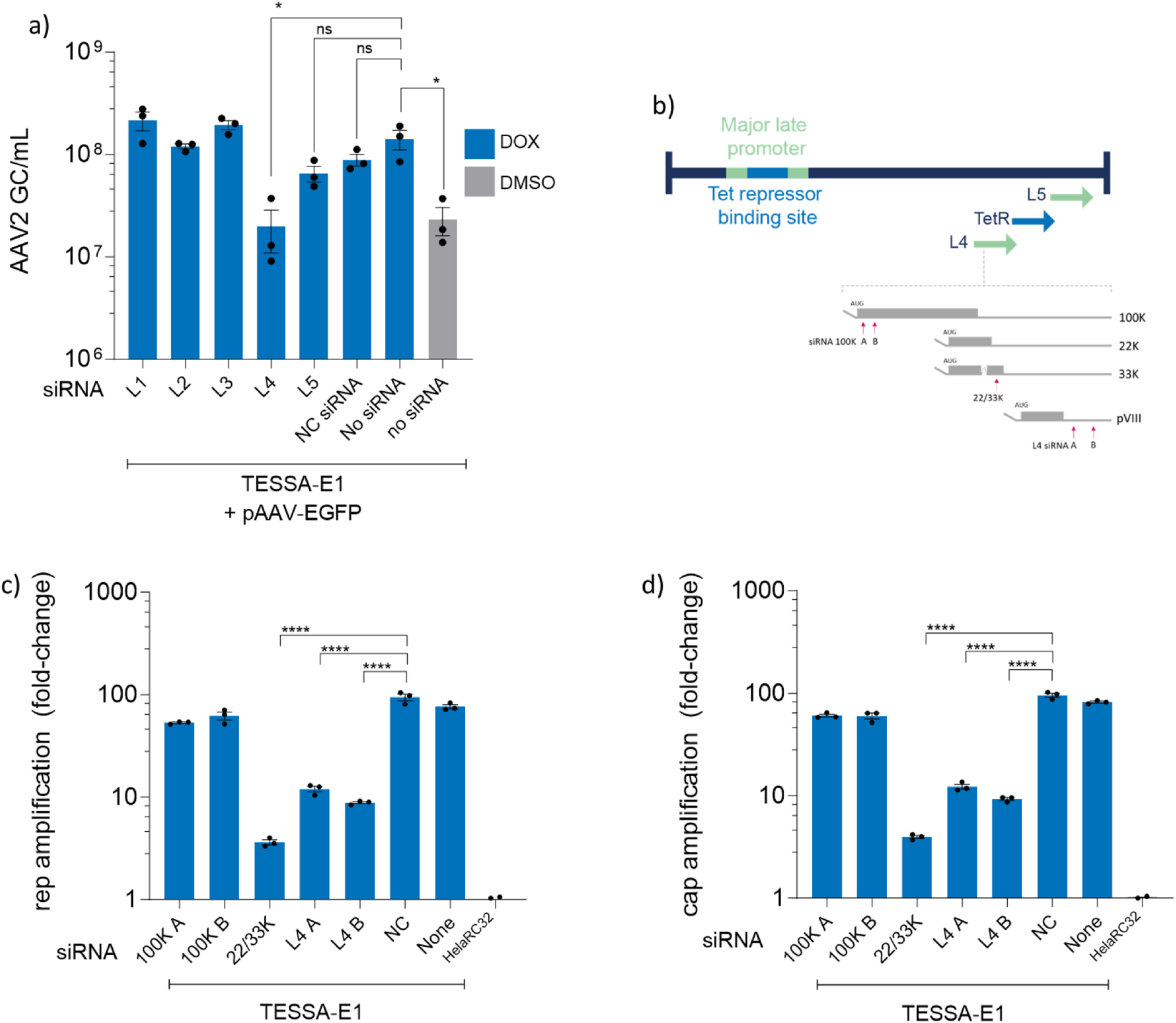
Transcription from the L4 22/33K unit is important for rAAV production from HeLaRC32 cells. (**a**) Production of rAAV2-EGFP in HelaRC32 cells transfected with siRNA targeting L1-L5 transcripts. siRNA-transfected HeLaRC32 cells were infected with TESSA-E1 (MOI of 50 TCID50/cell) and transfected with pAAV-EGFP and treated with doxycycline or DMSO. DNAse-resistant particles were quantified at 96 hpi by EGFP-specific qPCR. (**b**) Genome schematic of TESSA-E1 illustrating the L4 mRNA transcripts and complementary positions for siRNAs 100K A&B, 22/33K, and L4 A&B. Diagram not drawn to scale. HeLaRC32 cells transfected with the indicated siRNAs for 24 h, were infected with TESSA-E1 at an MOI of 50 TCID50/cell and transfected pAAV-EGFP. Cells were subsequently treated with doxycycline 0.5 µg/mL or DMSO as indicated. Total DNA was extracted at 72 hpi and quantified by (**c**) rep and (**d**) cap-specific qPCR. Data as mean ± SD of triple biological replicates. Analysed by one-way ANOVA followed by Tukey post hoc test. *p ≤ 0.05, ***p ≤ 0.0001.

To identify the missing factor(s) required to promote rep/cap amplification and rAAV production from TESSA-E1 during MLP repression, we employed an siRNA strategy targeting the mRNA transcripts from the MLP. Initially, HeLaRC32 cells were transfected with siRNAs that independently targets each of the Ad5 late family of transcripts (L1-L5), as the 3’ region of each mRNA transcript is shared within each family, prior to transfection with pAAV-EGFP and infection with TESSA-E1. Surprisingly, in the presence of doxycycline, knockdown of the L4 transcripts reduced rAAV yield to background levels observed from TESSA-E1 treated with DMSO (Fig. 3a). This suggested that the ‘missing’ CARE-replication factor(s) from TESSA-E1 was embedded within the L4 transcripts, which encodes for the adenovirus 100K, 22K, 33K and VIII protein. To identify the involvement of each of these proteins, we employed another siRNA strategy targeting different L4 transcripts. As shown in Fig. 3b, siRNAs were designed for specifically targeting L4 100K mRNA (100K A and 100K B), mRNA transcripts shared between L4 100K, 22K and 33K (22/33K), or an siRNAs that targets all L4 transcripts (L4 siRNA A and B). As expected, both siRNAs solely targeting the 100K mRNA did not affect amplification of (Fig. 3c) rep or (Fig. 3d) cap from the HeLaRC32 cells infected with TESSA-E1 and supplemented with doxycycline, which confirmed that the 100K protein was not essential for CARE amplification. Surprisingly, all siRNAs targeting knockdown of the 22/33K mRNA resulted in 9 to 20-fold decrease in rep and cap DNA compared to none-complementing (NC) siRNA. While the L4 A and B siRNAs targeted both 22/33K and the L4 VIII, it is unlikely that L4 VIII was involved as the siRNA solely targeting 22/33K resulted in the greatest reduction of rep/cap DNA in infected cells. This suggested that only the 22/33K proteins are necessary for CARE amplification.

### Expression of L4 22 and 33K *in trans* recovered rep/cap amplification in HeLaRC32 cells

**Figure 4:**
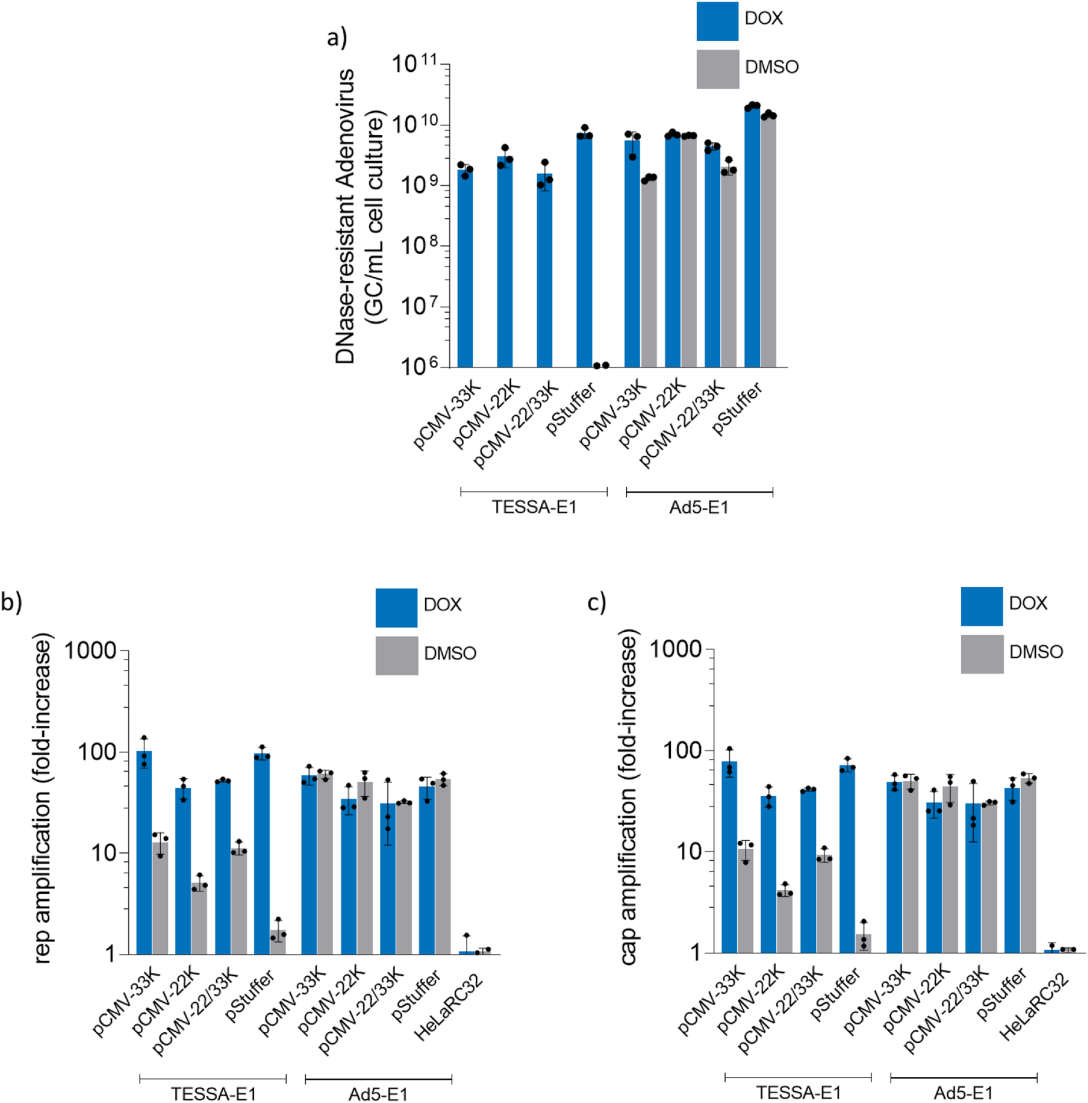
Expression of L4 22/33K *in trans* recovers rep/cap amplification from TESSA-E1. HeLaRC32 cells were transfected with the 22/33K expression plasmids (or stuffer DNA) and infected with TESSA-E1 or control Ad5-E1, and treated with DMSO or doxycycline 0.5µg/mL. At 72 hpi, each condition was harvested and DNAse-resistant particles were quantified by hexon-specific qPCR (**a**), or total DNA was extracted and CARE replication determined by qPCR using rep (**b**) and cap (**c**) primers and probes. Data presented as fold-change in rep and cap DNA compared to non-infected HeLaRC32 cells. Data as mean ± SD of triple biological replicates.

HeLaRC32 cells were transfected with plasmids expressing 22K, 33K or 22/33K (wherein the intron was retained) from the CMV promoter to assess whether they are able to recover rep/cap amplification from TESSA-E1. Since the 22K and 33K proteins have previously been implicated in activation of the MLP^18,19^, we initially confirmed that their expression *in trans* did not overcome TetR-repression of the MLP in TESSA-E1 by measuring adenoviral production in the presence of 22K and 33K. As shown in Fig. 4a, while a small decrease in TESSA-E1 (doxycycline group) and Ad5-E1 vectors was observed in HeLaRC32 cells transfected with the 22/33K expression plasmids compared to stuffer DNA, DNAse-resistant TESSA-E1 vectors remained undetected by adenovirus hexon-specific qPCR when doxycycline is absent. In addition, expression of both L4 22K and 33K resulted in a modest five- to tenfold increase in rep (Fig. 4b) and cap (Fig. 4c) DNA amplification from TESSA-E1, despite the absence of doxycycline. Overall, these results confirmed that repression of the MLP in TESSA-E1 remained intact during overexpression of 22/33K, and the 22/33K proteins are important for CARE-dependent rep/cap amplification.

### siRNA knockdown of Ad5 E2B recovered rep/cap amplification from TESSA-E1

As discussed previously, Krüger-Haag et al. showed that an adenovirus helper deleted of the 100K gene was unable to efficiently promote rep/cap amplification from an AAV packaging cell line^20^. Interestingly, the same authors showed that an adenovirus deleted of the pre-terminal protein (pTP) was fully capable of inducing rep/cap amplification and rAAV production. Adenovirus deleted for the pTP gene is incapable of DNA replication and shows undetectable levels of late mRNAs^20,22^. Specifically, the pTP is essential for initiation of adenovirus genome replication, a prerequisite for activation of the MLP and expression of late virus genes, including the 100K protein. As the pTP-deleted adenovirus is unable to induce MLP activity, it should therefore have only limited expression of the L4 genes from the L4P promoter, including the 22/33K and 100K, to support rep/cap amplification. This finding seems in stark contrast to our results which suggest that the L4 22/33K proteins are involved in rep/cap amplification and also with the inability of the 100K-deleted adenovirus to support this process.

**Figure 5:**
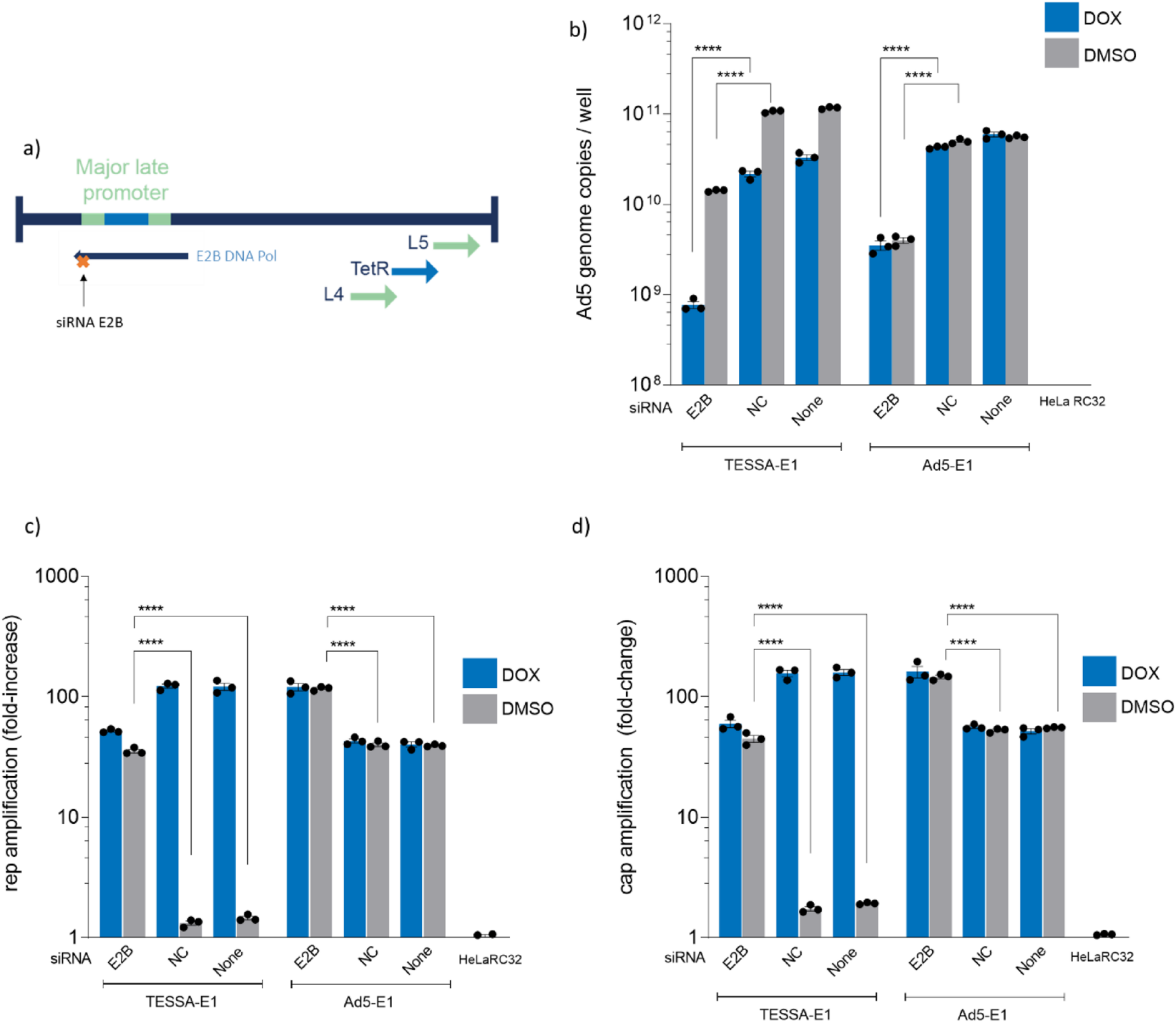
siRNA knockdown of Ad5 E2B transcription unit recovered rep and cap amplification in HeLaRC32 cells from TESSA-E1. (**a**) Schematic diagram illustrating complementary positions for knock-down of E2B DNA polymerase mRNA using siRNAs. HeLaRC32 cells transfected with siRNA targeting the Ad5 E2B unit, NC siRNA or mock transfection for 24 h, were infected with TESSA-E1 or Ad5-E1 at an MOI of 50 TCID50/cell. Cells were subsequently treated with doxycycline or DMSO. Total DNA was extracted at 72 hpi and quantified by (**b**) hexon, (**c**) AAV2 rep, and (**d**) AAV2 cap-specific qPCR. Data as mean ± SD of triple biological replicates. Analysed by one-way ANOVA followed by Tukey post hoc test. ****p ≤ 0.0001.

To reconcile this discrepancy, an obvious biological difference between the 100K-deleted adenovirus and TESSA-E1 in the absence of doxycycline (unable to support rAAV production from stable packaging cells) and the pTP-deleted adenovirus (able to support rAAV production from stable packaging cells) is that the formers viruses are able to undergo adenovirus genome replication during an ongoing infection. This led us to hypothesize that low levels of 22K/33K produced from the L4P may be enough to support rep/cap amplification and rAAV replication, but can be sequestered by large amounts of adenovirus genomes replicated by TESSA-E1 or a 100K-deleted adenovirus. To test this hypothesis, we employed an siRNA strategy to reduce DNA replication of TESSA-E1 by knockdown of the E2B transcription unit encoding the virus DNA polymerase and pTP (Fig. 5a) to assess whether it enables recovery of rep/cap DNA amplification^23^. As shown in Fig. 5b, while we observed a fivefold increase in DNA replication from TESSA-E1 during MLP repression in the absence of doxycycline, consistent with our previous findings^15^, a significant tenfold reduction in adenovirus genomes were detected in HeLaRC32 cells transfected with E2B targeting siRNA. A similar tenfold reduction in adenovirus genomes was also observed from E2B siRNA knockdown of the Ad5-E1 control. Surprisingly, in the absence of doxycycline, despite a modest tenfold reduction in TESSA-E1 genomes from E2B siRNA knockdown, a 30-fold increase in rep (Fig. 5c) and cap (Fig. 5d) DNA amplification was observed, confirming a direct correlation between the levels of adenoviral genomes and CARE-amplification within the cell. Furthermore, knockdown with E2B siRNA also promoted a threefold increase in rep and cap DNA in cells infected with the control Ad5-E1 compared to NC and no siRNA, suggesting rep/cap DNA and rAAV replication can also be enhanced by a reduction of adenoviral genomes.

### Temperature-sensitive DNA polymerase mutant TESSA-E1-tsDNA enables recovery of rep/cap amplification in HeLaRC32 cells

**Figure 6:**
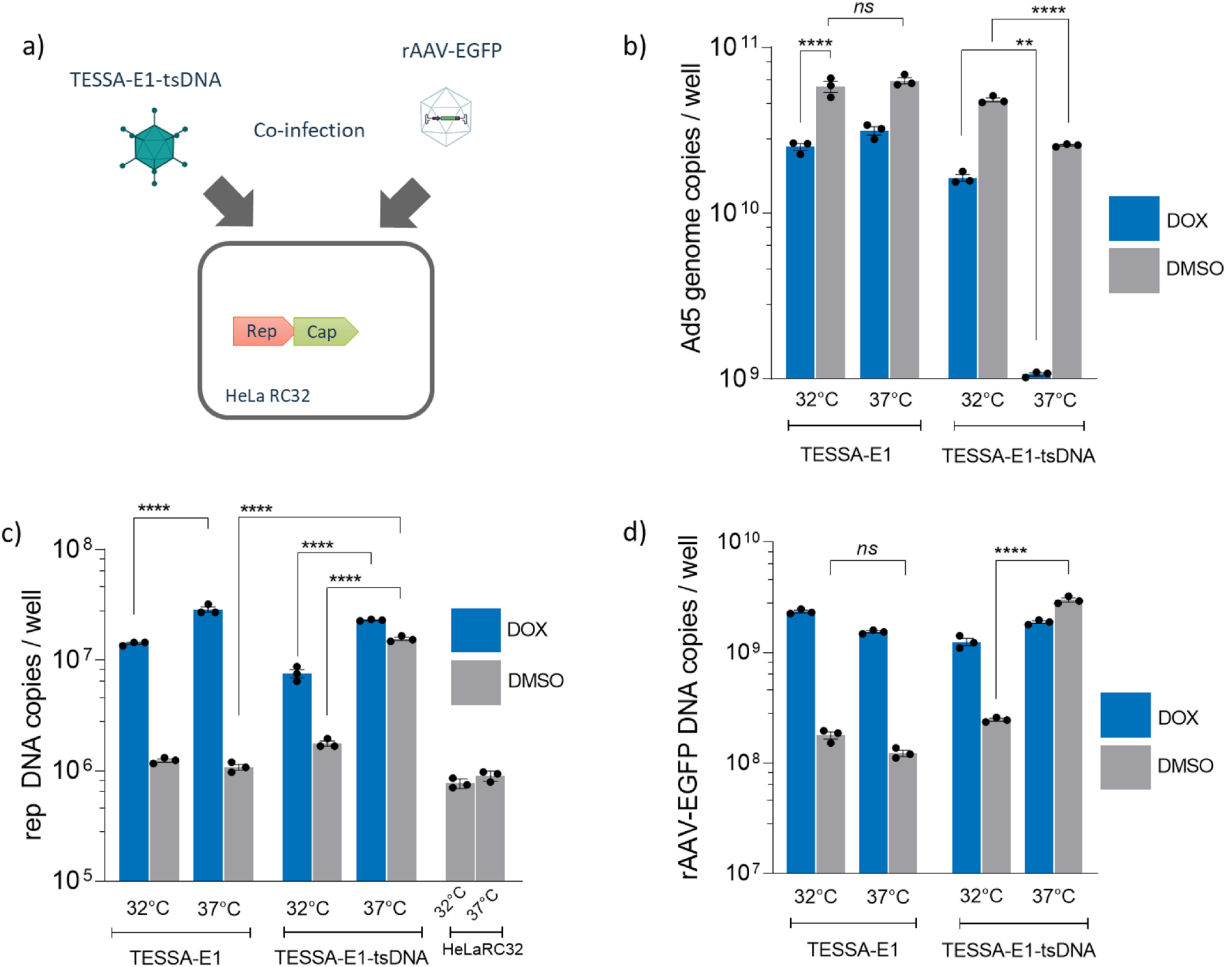
Temperature-sensitive DNA polymerase mutant TESSA-E1-tsDNA enables recovery of rep and cap amplification in HeLaRC32 cells. (**a**) Schematic diagram for production of rAAV2 from HeLaRC32 cells. Cells were co-infected using rAAV2-EGFP (MOI of 100 GC/cell) with TESSA-E1 or TESSA-E1-tsDNA and cultured with DMSO or doxycycline. Cells were incubated at 32 °C and 37 °C. Total DNA was extracted at 72 hpi and quantified by (**b**) hexon, (**c**) AAV2 rep, and (**d**) EGFP-specific qPCR. Data as mean ± SD of triple biological replicates. Analysed by one-way ANOVA followed by Tukey post hoc test. ****p ≤ 0.0001.

Adenovirus can be engineered with a temperature-sensitive switch (*ts36*) within the virus DNA polymerase, meaning that the mutant-*ts36* DNA polymerase is fully active at 32°C but activity is significantly reduced at 37 °C^24–27^. This modification was introduced into TESSA-E1, and the resulting TESSA-E1-tsDNA was infected into HeLaRC32 cells alongside transfection with plasmid pAAV-EGFP (Fig. 6a). In measuring the production of TESSA-E1-tsDNA (adenovirus) genomes, as predicted significantly fewer genomes were produced at 37 °C compared to 32 °C both in the presence of doxycycline and in its absence (Fig. 6b). Intriguingly, for both regular TESSA-E1 and the temperature-sensitive variant, more genomes were made in the absence of doxycycline at both temperatures, a phenomenon seen earlier and presumably reflecting the fewer cellular resources being deployed into transcription in the absence of doxycycline, because the MLP is not activated. Against this background we were testing the hypothesis that AAV rep and cap genes would be amplified in the absence of MLP activation (i.e. without doxycycline) when adenovirus genome replication was impaired, i.e. using the TESSA-E1-tsDNA at 37 °C and not at 32 °C. Our data support this hypothesis, with the temperature-based inhibition of E2B transcription yielding at least tenfold increases in CARE-dependent amplification (Fig. 6c) and replication of rAAV-EGFP, demonstrating successful rAAV genome replication (Fig. 6d). Taken altogether these data suggest that sufficient 22/33K proteins are made to enable CARE amplification even without MLP activation, provided that adenovirus genome replication is limited.

## Discussion

While TESSA was fully capable of providing helper functions to enable rAAV production when the rep and cap genes were provided *in trans* via plasmid transfection or from a TESSA vector encoding these genes^15^, we found that in AAV packaging HeLaRC32 cells TESSA-E1 was unable to support rep/cap DNA amplification and rAAV production when its MLP was repressed. Through a series of siRNA and complementation assays, we showed that the adenovirus intermediate-late proteins 22K and 33K were essential for CARE-dependent rep/cap DNA amplification.

As Kruger-Haag et al. showed that while the 100K-deleted adenovirus was unable to support rAAV production, an observation consistent with TESSA-E1 during MLP repression, the pTP-deleted adenovirus successfully promoted rep/cap DNA amplification despite this pTP-mutant being unable to undergo DNA replication with concomitant poor MLP activation and only limited 22/33K^20^. This apparently contradictory finding led us to hypothesize that, perhaps, sufficient 22/33K proteins are expressed from its internal L4P but can be titrated away from the CARE-amplification process by large amounts of replicating adenovirus genomes within the cell. Indeed, upon siRNA knockdown of the E2B transcription unit, encoding for the virus DNA polymerase and pTP, we were able to recover rep/cap DNA amplification from TESSA-E1 during MLP repression. This result confirmed our initial hypothesis and also provide a mechanistic explanation for the inability of the 100K-deleted adenoviruses to support rAAV production from stable AAV packaging cell lines.

The precise molecular role of the 22K/33K proteins currently remains uncertain, including how their coding sequences are involved in amplification of AAV rep/cap genes from stable producer cells. While we showed that expression of 22K/33K proteins *in trans* from expression plasmids resulted in a modest five- to tenfold increase in rep and cap DNA amplification from TESSA-E1, despite the absence of doxycycline, it did not fully recover to levels observed from the doxycycline group. Possibly, temporal expression of 22K/33K proteins are required for optimal amplification of AAV rep/cap, as their expression in situ are tightly controlled from the adenovirus^18,19^. Additionally, expression of the 22K or 33K from the CMV expression plasmids both resulted in AAV rep/cap amplification may suggest functional redundancies between these proteins. The L4-22K and -33K proteins, both sharing the first 105 amino acids but differing towards the C-terminus, are multifunctional and involved in regulating the expression of early and late adenoviral genes, RNA splicing and the genome encapsidation process^16^. While the L4-22K protein was shown to bind A repeats in the adenovirus packaging signal at a conserved TTTG motif, L4-33K interacts with the IVa2 protein that associates with the packaging domain of the Ad genome and functions as DNA packaging ATPase^28,29^. It was also shown by co-immunoprecipitation assays and confocal-microscopy that the L4-33K protein co-localizes with adenovirus DBP, which was reported to be essential for rep/cap DNA amplification from AAV packaging cells^12,30^. Possibly, physical binding of 22/33K proteins to the adenovirus genome limits their availability required for the CARE-amplification process.

To enable the use of TESSA-E1 for production of rAAV from stable rep/cap packaging cells, we introduced the temperature-sensitive DNA polymerase mutation *ts36* to generate TESSA-E1-tsDNA. This helper TESSA-E1-tsDNA is effectively produced in HEK293 cells at 32 °C and supplemented with doxycycline, to enable full activity of the *ts36* polymerase and whilst inhibiting activity of the TetR for full MLP functionality, respectively. While replication of TESSA-E1-tsDNA genomes was reduced by 15-fold at the non-permissive temperature of 37 °C (with doxycycline), a modest twofold reduction in TESSA-E1-tsDNA genomes was observed in the absence of doxycycline likely due to the increase efficiency of adenoviral genome replication observed from repression of the MLP^15^. Interestingly, at 37 °C, the temperature used for rAAV production, a modest reduction in the replication of TESSA-E1-tsDNA significantly recovered amplification of the rep/cap gene for rAAV replication in HeLaRC32 cells. We propose TESSA-E1-tsDNA as an efficient helper virus for contaminant-free manufacture of rAAV from stable rAAV packaging cell lines. Future development may explore approaches to further repress TESSA-E1-tsDNA genome replication by employing expression of shRNA, small RNAs, or MLP-TSS-sRNA shown to inhibit adenovirus, or heterologous expression of 22/33K to enhance rAAV production from stable AAV packaging cells^31^. In addition to recent novel approaches designed to improve rAAV production, and as a DNA delivery vector, this range of options should provide a raft of strategies for efficient scalable manufacture of all AAV serotypes using stable packaging cells, avoiding contamination issues and dramatically decreasing the associated costs^32–35^.

## Methods

### Virus generation and titration

Construction of the TESSA genome plasmid (pSU390) has been described previously (Su et al. 2022). To generate TESSA-E1 and the control Ad5-E1 plasmids, the Ad5 E1 coding sequence was amplified by PCR from plasmid encoding the wildtype Ad5 genome (T38, OXGENE) using forward primer 5’-GACGGCGATCGCTGGGGCGGCCGCCATCGATGGGTTAATTAATGTAGTGTATTTATACCCGG’-3 and reverse primer 5’-GACCCCCACCTTATATATTCTTTCCCACCCTTAATTAAGCCACGCCCACACATTTCAG TACCTC-3’ and inserted into pSU390 and OG268 plasmids linearised with PacI via Gibson DNA assembly (New England Biolabs, MA, USA), to generate TESSA-E1 (pSU708) and Ad5-E1 plasmids (pSU704), respectively. For incorporating the *ts36* mutation into the DNA polymerase coding sequence of TESSA-E1, a shuttle plasmid (pSU180), which encodes a portion of the Ad5 E2B polymerase fragment, was used as a template for PCR using forward primer 5’- CTGCACGTATTCGCGCG -3’ with reverse primer 5’- CTCAGCTCCCCTGAAGAGtttACCTACGAGGAACTT -3’, and forward primer 5’- CCCACAATGTAAAaTTCCAAGAAGC-3’ with reverse primer 5’- AAGTTCCTCGTAGGTaaaCTCTTCAGGGGAGCTGAG -3’, to generate two DNA fragments incorporating the *ts36* mutation. These fragments were inserted into DraIII and BsrGI (New England Biolabs, MA, USA) linearized pSU180 via Gibson DNA assembly, generating plasmid pSU1278. The ts36-E2B fragment was subsequently excised from pSU1278 using SphI and NheI (New England Biolabs, MA, USA) and inserted into pSU390 (linearized with XbaI and BstZ17I) via Gibson DNA assembly to generate pSU1294. The Ad5 E1 genes were extracted from pSU708 using AsiSI and BstZ17I (New England Biolabs, MA, USA) and inserted into PacI-linearized pSU1294 via Gibson DNA assembly, generating TESSA-E1-tsDNA (pSU1316). Positive clones were isolated and virus recovery, and infectious titration, carried out in HEK293 cells as previously described^15^.

### Determining intact adenovirus particles by PicoGreen assay

Intact adenovirus particles in purified stocks were determined using the PicoGreen dsDNA assay (Invitrogen, UK) which uses a fluorescent nucleic acid stain specific for double-stranded DNA. Purified viral stocks were diluted in TE buffer containing 0.1% SDS, 25mM EDTA and incubated at 37 °C for 15 min to disassociate the virus capsid. Samples and the standard curve generated using bacteriophage lambda DNA were measured using the PicoGreen dsDNA assay kit according to the manufacturer’s instructions. Concentration of intact particles was calculated based upon 1 μg is equivalent to 2.7 × 10^10^ genome intact particles^36^. The ratio of intact adenoviral particles, determined by the PicoGreen assay, and the Tissue Culture Infectious Dose (TCID50) is routinely determined to be < 20:1.

### Cell culture

HEK293 cells and HeLaRC32 cells were purchased from Cell Biolabs (AD-100, CA, USA) and ATCC (CRL-2972, VA, USA), respectively, and cultured in Dulbecco’s Modified Eagle Medium (DMEM; Sigma-Aldrich, MO, USA) supplemented with 10% (v/v) heat-inactivated foetal bovine serum (FBS; Gibco, MA, USA). Cell lines were maintained at 5% CO_2_, 37 °C, and 95% humidity.

### Plasmids and siRNA transfection

siRNA transfections were performed using Lipofectamine RNAiMAX (ThermoFisher, USA) according to the manufacturer’s protocol. Reverse transfection of HEK293 cells with siRNA was carried out in 48-well plate format. For each well, 3 pmol of siRNA diluted in 30 μL of OptiMEM (Life Technologies, UK) was complexed with 1 μL of RNAiMAX suspended OptiMEM. Reaction was incubated at ambient temperature for 10 min before adding to the well. Subsequently, 9 × 10^4^ cells per well suspended in DMEM containing 10% FBS was added to each well and the plate was transferred to a 37°C incubator. After 24 h, cells were infected with adenovirus vectors as indicated in the experiments. siRNA sequences are provided in Supplementary Table S1. All CMV expression plasmids were provided by OXGENE (Oxford, UK) and the pAAV-EGFP was described previously^15^. Cells were seeded at 9 × 10^4^ cells per well for 24 h and each well was transfected with 750 ng of plasmid DNA complexed using linear PEI 25kDA (Polysciences) at a 1:3 DNA to PEI mass ratio.

### Quantification of viral genomes using qPCR

For quantification of total adenovirus genomes in HeLaRC32 cells, total DNA was extracted from culture media and cellular lysates using DNeasy Blood and Tissue kit (Qiagen, Venlo, Netherlands). Five microlitres of DNA eluent were used in qPCR reactions using TaqMan Fast Advanced Master Mix (Applied Biosystems, CA, USA) in a StepOnePlus Real-Time PCR System (Applied Biosystems, CA, USA). Primer sequences for targeting Ad5 hexon are forward 5′-CACTCATATTTCTTACATGCCCACTATT-3′, reverse 5′- GGCCTGTTGGGCATAGATTG-3′ and TaqMan probe 5′-AGGAAGGTAAC TCACGAGAACTAATGGGCCA-3′. Primer sequences targeting the EGFP are forward 5′-GAACCGCATCGAGCTGAA-3’, reverse 5′-TGCTTGTCGGCCATGATATAG-3′, and TaqMan probe 5′-ATCGACTTCAAGGAGGACGGCAAC-3′. Primer sequences for targeting AAV2 rep forward 5′-GGCCTCATACATCTCCTTCAAT-3′, reverse 5′- AGTCAGGCTCATAATCTTTCCC-3′ and TaqMan probe 5′- TCCAACTCGCGGTCCCAAATCAA-3′. Primer sequences for targeting AAV2 cap are forward 5′-CGACCCAAGAGACTCAACTTC-3′, reverse 5′-GAACCGTGCTGGTAAGGTTAT-3′ and TaqMan probe 5′-AAAGAGGTCACGCAGAATGACGGT-3′. PCR cycles were as follows: 95 °C 10 min; 40 times (95 °C 1 s, 60 °C 20 s). Extraction and quantification of DNAse-resistant rAAV genomes were carried out as previously described^15^.

### Statistical analysis

Data presented as mean ± standard deviation (SD), unless otherwise stated. Significance evaluated using one-way ANOVA followed by Tukey post hoc test, unless otherwise stated, and denoted on the graphs as *P ≤ 0.05 **P ≤ 0.01, ***P ≤ 0.001, ****P ≤ 0.0001.

### Supplementary Information

Supplementary Information 1.

Supplementary Information 2.

## Data Availability

All data generated or analysed during this study are included in this published article [and its supplementary information files]. Plasmids and viral vectors generated from this study are available upon reasonable requests. Viral vectors, plasmid materials and datasets can be requested from the corresponding author W.S.
