# Supplementary material for: AAV production in stable packaging cells requires expression of adenovirus 22/33K protein to allow episomal amplification of integrated rep/cap genes
Source: Sci Rep. 2023 Dec 7;13:21670. doi: 10.1038/s41598-023-48901-z (PMC10709602; doi:10.1038/s41598-023-48901-z)
Supplement: Supplementary file 2 — Supplementary Information 2. [file 41598_2023_48901_MOESM2_ESM.pdf]

Supplementary

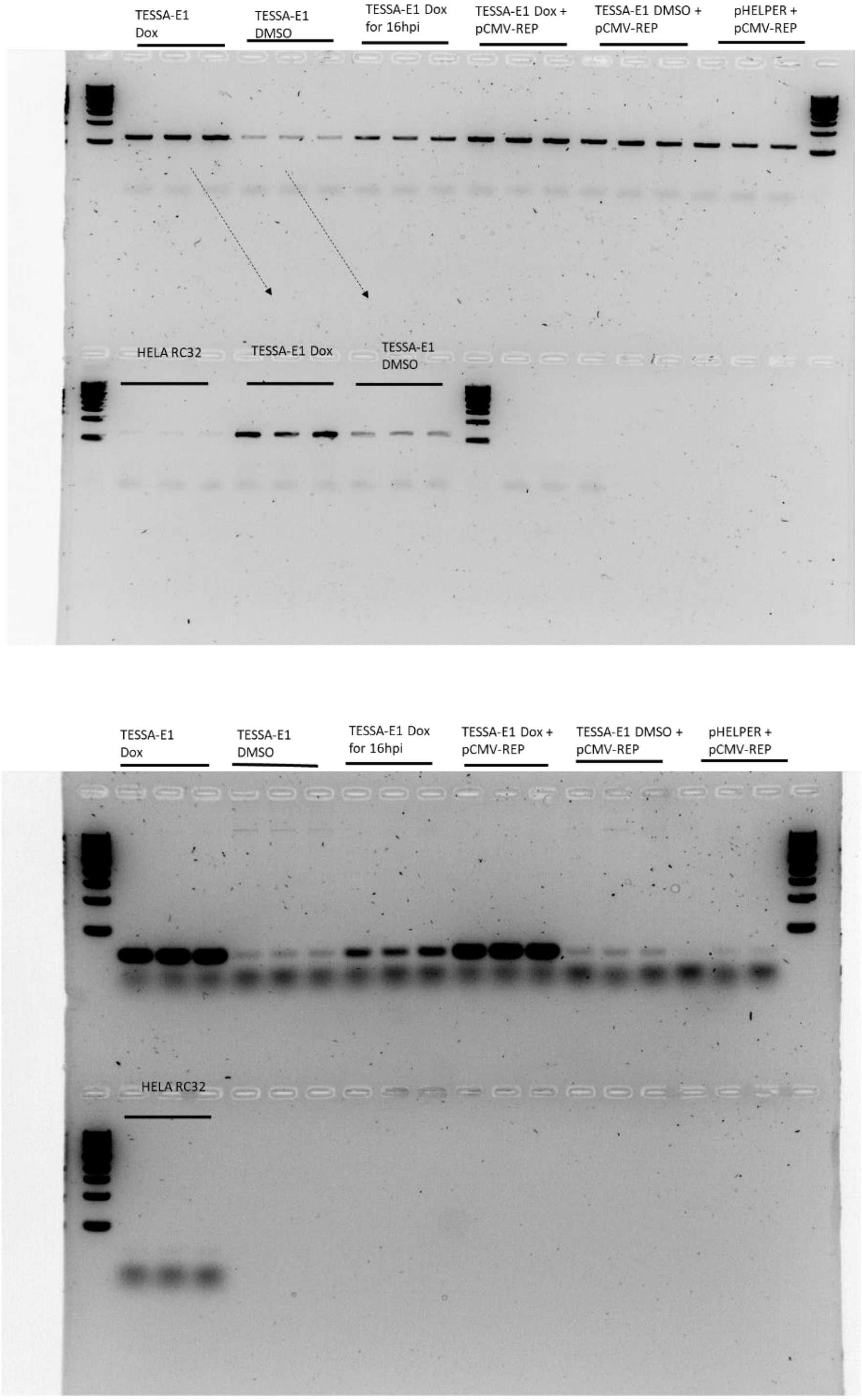

Supplementary figure 1. Episomal DNA amplification in HeLaRC32 cells. Cells were infected with Ad5-E1 or TESSA-E1 and cultured with DMSO or doxycycline and used for PCR with AAV2 rep (top blot) and cap2-specific (bottom blot) primers and resolved by gel electrophoresis.

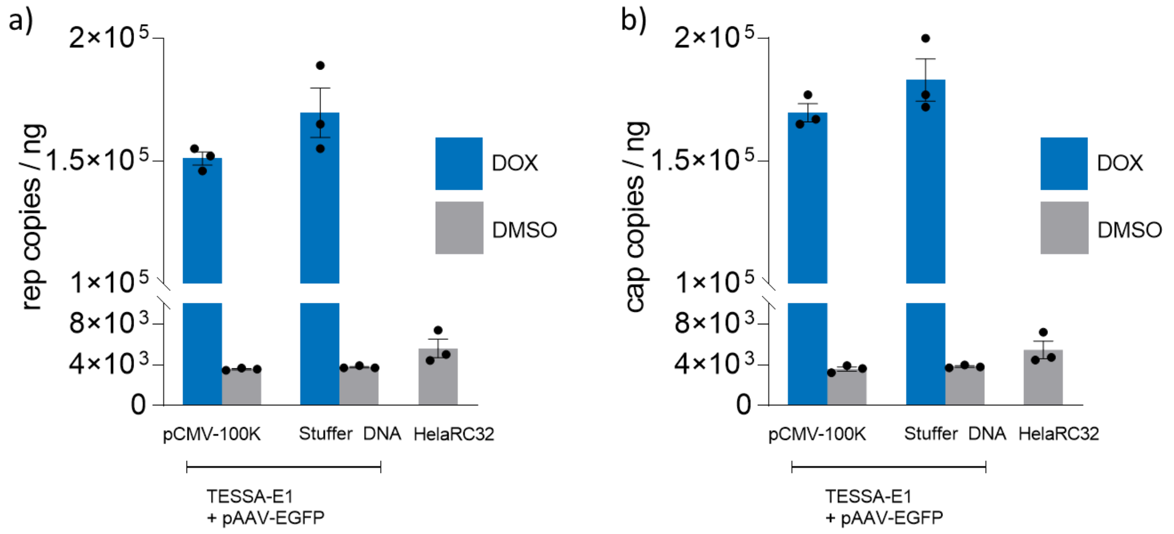

Supplementary figure 2. Quantification of AAV2 rep and cap in TESSA-E1 infected (MOI50) HeLaRC32 cells that were transfected with the pCMV-100K or stuffer DNA. Total DNA was extracted at 96 hpi and quantified by (a) rep or (b) cap-specific qPCR. Data presented as copies per ng of DNA. Data presented as mean ± SD of triple biological replicates.

Supplementary table 1. Sequences of siRNAs used in this study.

**Name**	**Sense**	**Antisense**	**Origin**
Ad5 Pol	CAACGUCUUCCAGCGUCCAACCAUA	UAUGGUUGGACGCUGGAAGACGUUG	[23]
100K A	CAGUACCAACAGAGGAUAAAAAGCA	UGCUUUUUAUCCUCUGUUGGUACUGAG	Custom
100K B	GUACCAACAGAGGAUAAAAAGCAAG	CUUGCUUUUUAUCCUCUGUUGGUACUG	Custom
22/33K	GAACAAGAGCUGAAAAUAAAAAACA	UGUUUUUUAUUUUCAGCUCUUGUUCUU	Custom
L4 A	ACCUUGGAUUACAUCAAGAUCAUCT	AGAUGAUCUUGAUGUAAUCCAAGGUUA	Custom
L4 B	AACUGUCCUAACCUUGGAUUACATC	CGUUGACAGGAUUGGAACCUAAUGUAG	Custom
Ad5 L1	AUGCUAGUAGCCCAUUUCCAAGCTT	UCUACGAUCAUCGGGUAAAGGUUCGAA	Custom
Ad5 L2	AACAAGUUGCAUGUGGAAAAAUCAA	UUUUGUUCAACGUACACCUUUUUAGUU	Custom
Ad5 L3	GUCACUUGAAAAACAUGUAAAAATA	AACAGUGAACUUUUUGUACAUUUUUAU	Custom
Ad5 L4	ACCUUGGAUUACAUCAAGAUCAUCT	AUUGGAACCUAAUGUAGUUCUAGUAGA	Custom
Ad5 L5	GCAGUCAAAUACUUGCUACAGUUTC	ACCGUCAGUUUAUGAACGAUGUCAAAG	Custom
